# Supplementary material for: Elevated Plasma Vitamin B12 in Patients with Hepatic Glycogen Storage Diseases
Source: J Clin Med. 2020 Jul 22;9(8):2326. doi: 10.3390/jcm9082326 (PMC7463656; doi:10.3390/jcm9082326)
Supplement: Supplementary file 1 [file jcm-09-02326-s001.pdf]

Supplemental Information for:

Elevated Plasma Vitamin B_{12} in Patients with Hepatic Glycogen Storage Diseases

Julia Hinkel [1], Johannes Schmitt [1], Michael Wurm [1,2], Stefanie Rosenbaum-Fabian [1], Karl Otfried Schwab [1], Donald W. Jacobsen [3], Ute Spiekerkoetter [1], Sergey N. Fedosov [4], Luciana Hannibal [5,*,†] and Sarah C. Grünert [1,*,†]

1 Department of General Pediatrics, Adolescent Medicine and Neonatology, Medical Center - University of Freiburg, Faculty of Medicine, 79106 Freiburg, Germany; julia.hinkel@uniklinik-freiburg.de (J.H.); johannes.schmitt@uniklinik-freiburg.de (J.S.); stefanie.rosenbaum-fabian@uniklinik-freiburg.de (S.R.-F.); karl.otfried.schwab@uniklinik-freiburg.de (K.O.S.); ute.spiekerkoetter@uniklinik-freiburg.de (U.S.)
2 Department of Pediatrics, St. Hedwigs Campus, University Children's Hospital Regensburg, 93049 Regensburg, Germany; Michael.Wurm@barmherzige-regensburg.de (M.W.)
3 Department of Cardiovascular and Metabolic Sciences, Lerner Research Institute, Cleveland Clinic, 44195 Cleveland, Ohio, USA; jacobsd@ccf.org
4 Department of Molecular Biology and Genetics, Aarhus University, DK-8000 Aarhus C, Denmark; snf@mbg.au.dk
5 Laboratory of Clinical Biochemistry and Metabolism, Department of General Pediatrics, Adolescent Medicine and Neonatology, Medical Center - University of Freiburg, Faculty of Medicine, 79106 Freiburg, Germany
* Correspondence: luciana.hannibal@uniklinik-freiburg.de (L.H.); sarah.gruenert@uniklinik-freiburg.de (S.C.G.)
† Equal senior contribution.

Figure S1. Questionnaire employed to assess dietary intake of vitamin B12 in GSD patients.

Figure S2. Correlation between vitamin B_{12} and triglycerides concentrations in plasma.

Figure S3. Correlation between vitamin B_{12} concentrations and liver transaminases in plasma.

Figure S4. Biochemical parameters of healthy controls and GSD patients.

Figure S5. Correlation between estimated daily intake of vitamin B_{12} and plasma concentration of vitamin B_{12}.

Table S1. Results of Spearman correlation analysis using log10-transformed datasets.

Vitamin B12-Intake

NAME ______________________________

Please provide information on the frequency of consumption of the following foods:

	**daily**	**3-6x / week**	**1-2x / week**	**rarely**	**never**
MEAT					
- cold cuts/ sausage	◻	◻	◻	◻	◻
- pork,beef, venison	◻	◻	◻	◻	◻
- poultry	◻	◻	◻	◻	◻
- offal	◻	◻	◻	◻	◻
FISH	◻	◻	◻	◻	◻
MILK PRODUCTS	◻	◻	◻	◻	◻
EGGS	◻	◻	◻	◻	◻

Please provide information on food supplements below:

Do you take dietary supplements, minerals or other products that can be purchased in pharmacies, supermarkets or drugstores?

PRODUCT	**daily**	**3-6x / week**	**1-2x / week**	**rarely**	**never**
____________	◻	◻	◻	◻	◻
____________	◻	◻	◻	◻	◻
____________	◻	◻	◻	◻	◻
____________	◻	◻	◻	◻	◻
____________	◻	◻	◻	◻	◻
____________	◻	◻	◻	◻	◻

Figure S1. Questionnaire employed to assess dietary intake of vitamin B12 in GSD patients.

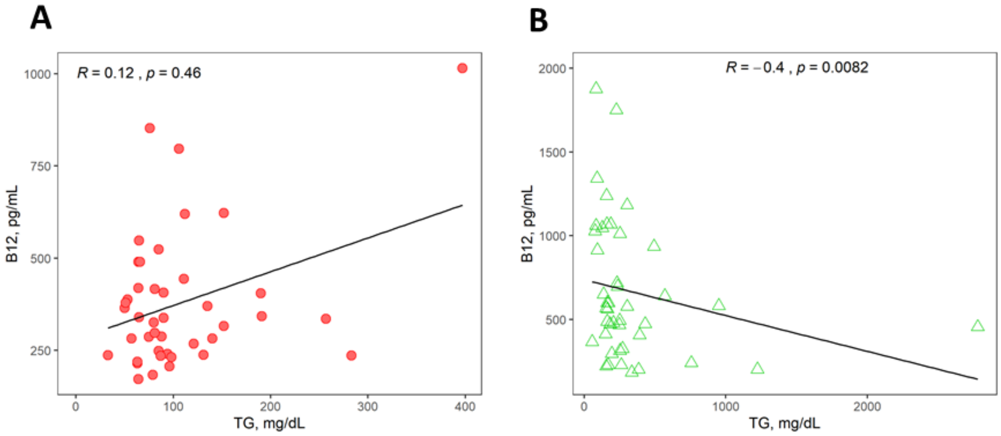

Figure S2. Correlation between vitamin B_{12} and triglycerides concentrations in plasma. Panel A) Triglyceride and vitamin B_{12} concentration in healthy controls. Panel B) Triglyceride and vitamin B_{12} concentration in GSD patients. A significant correlation was only observed in the GSD patient group ($p = 0.0082$).

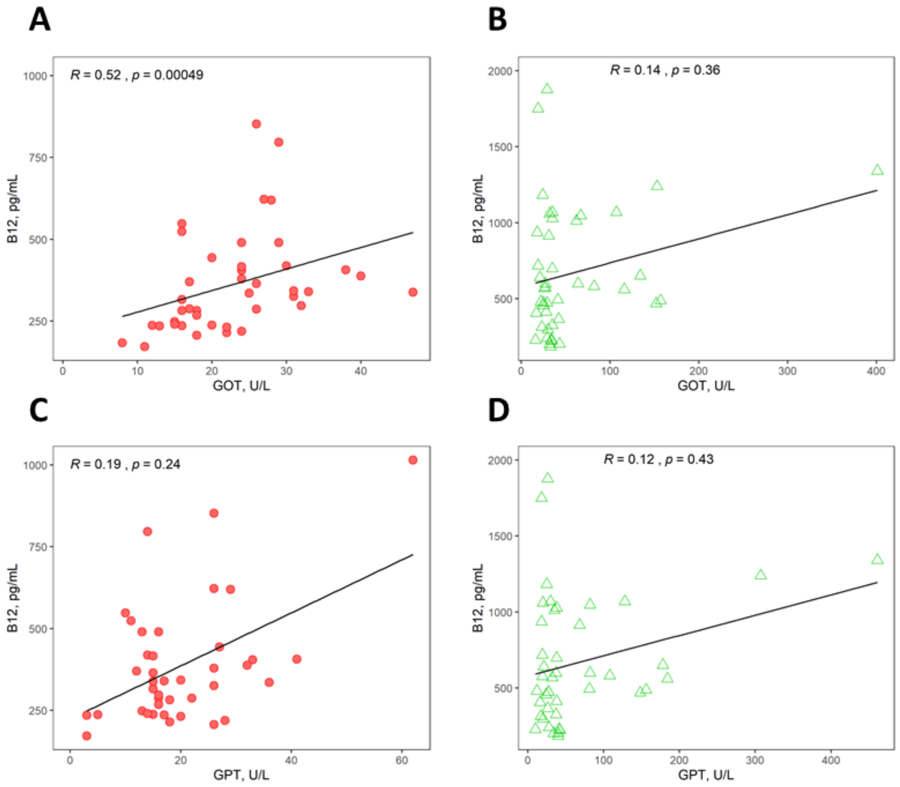

Figure S3. Correlation between vitamin B_{12} concentrations and liver transaminases in plasma. Panel A) Correlation between vitamin B_{12} and GOT (ASAT) in healthy controls. Panel B) Correlation between vitamin B_{12} and GOT (ASAT) in GSD patients. Panel C) Correlation between vitamin B_{12} and GPT (ALAT) in healthy controls. Panel D) Correlation between vitamin B_{12} and GPT (ALAT) in GSD patients. A significant correlation was only observed for B_{12} versus GOT in the healthy control group (p = 0.00049).

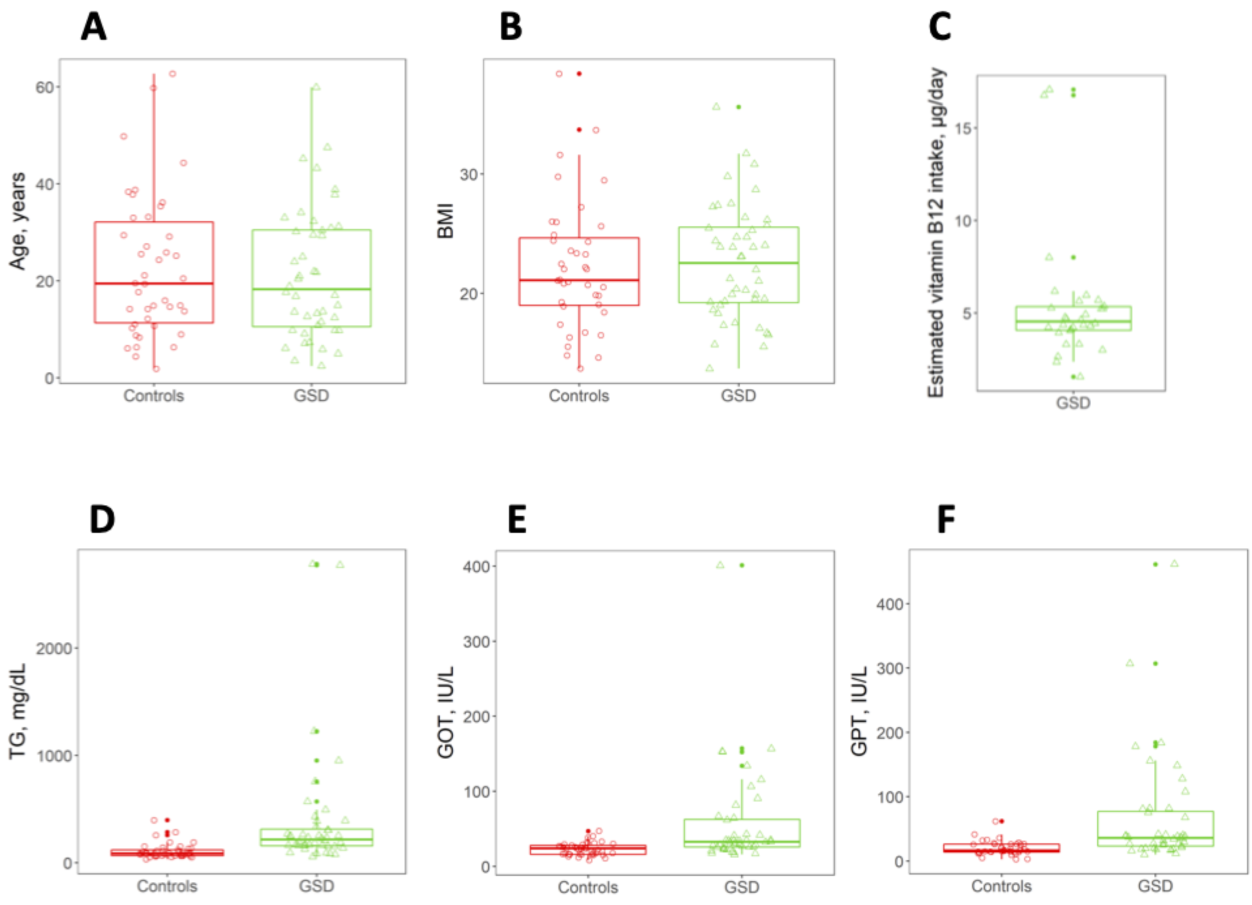

Figure S4. Biochemical parameters of healthy controls and GSD patients. Panels A to F present data for age, BMI, estimated vitamin B_{12} intake, TG, GOT and GPT, respectively. Estimated vitamin B_{12} intake was not available for the healthy control group (Panel C). Statistically significant differences were identified for triglycerides (TG), plasma vitamin B_{12}, GOT and GPT. Mean values, concentration ranges and p values are provided in Table 1 in the main manuscript.

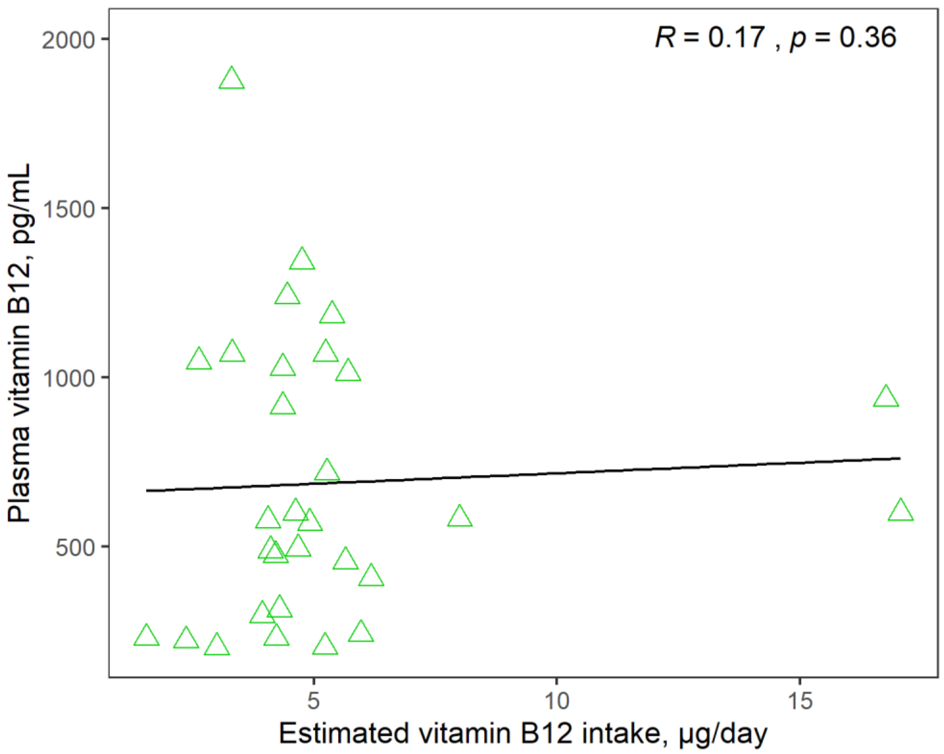

Figure S5. Correlation between estimated daily intake of vitamin B_{12} and plasma concentration of vitamin B_{12}. A total of 30 out of the 32 GSD patients for whom estimated vitamin B_{12} intake data were available were included in the analysis. Of the total of 32 GSD patients with intake data available, two were excluded: one patient was excluded because vitamin B_{12} concentration in plasma could not be measured. A second GSD patient was excluded due to an excessive supplementation with vitamin B_{12} of 1 mg per day. No statistically significant correlation was found between estimated dietary intake and plasma concentration of vitamin B_{12}.

Table S1. Results of Spearman correlation analysis using log10-transformed datasets

	Rho (R) value (95% correlation coefficient)	p value
	**log10 GOT vs. log10 B_{12}**	
Healthy controls	0.50 (0.23 to 0.70)	<0.001
GSD patients	0.14 (-0.17 to 0.43)	0.356
	**log10 GPT vs. log10 B_{12}**	
Healthy controls	0.19 (-0.14 to 0.48)	0.243
GSD patients	0.12 (-0.19 to 0.41)	0.435
	**log10 Triglycerides vs. log10 B_{12}**	
Healthy controls	0.12 (-0.20 to 0.41)	0.46
GSD patients	-0.40 (-0.63 to -0.10)	0.008
